# Finite element analysis of flexural behavior of UHPC-strengthened RC beams based on experimental calibration

**DOI:** 10.1038/s41598-026-50034-y

**Published:** 2026-05-09

**Authors:** Jiawei Wang, Zhongbin Li

**Affiliations:** 1 Engineering Research Center of Anhui Green Building and Digital Construction, Anhui Polytechnic University, Wuhu, 241000 China; 2 School of Architecture and Civil Engineering, Anhui Polytechnic University, Wuhu, 241000 China

**Keywords:** Flexural strengthening of RC beams, Ultra-high performance concrete, DIC technology, UHPC-to-concrete bond behavior, Finite element analysis, Engineering, Materials science

## Abstract

To enhance the strengthening effectiveness of existing reinforced concrete (RC) beams and fully utilize the material properties of ultra-high performance concrete (UHPC), this study systematically investigates the flexural strengthening mechanism of UHPC layers through mechanical property tests, four-point bending experiments, and refined finite element simulations. The research first calibrated the constitutive model of UHPC via uniaxial tension and compression tests. Subsequently, flexural tests were conducted on RC beams strengthened with a 40 mm thick UHPC layer, and the crack propagation process was observed using digital image correlation (DIC) technology. Based on experimental results, a finite element model considering material nonlinearity and interfacial bond-slip behavior was established. Parameters such as the thickness of the strengthening layer, material strength, and reinforcement ratio were analyzed. The results demonstrate that the UHPC strengthening layer significantly improves the flexural capacity, stiffness, and crack resistance of RC beams. The reinforced UHPC strengthening layer exhibited even more pronounced enhancements, with the cracking load and ultimate load increasing by up to 145.2% and 67.9%, respectively. DIC results revealed that cracks initiated in the UHPC layer and propagated across the interface into the RC beam. Surface roughening treatment ensured effective composite action without debonding failure. Finite element analysis further indicated that unreinforced strengthening layers led to stress concentration at the beam bottom, exhibiting brittle failure characteristics. In contrast, reinforced strengthening layers significantly improved stress distribution, resulting in more uniform and delayed crack development. The load–displacement curves displayed a distinct plateau after the peak load, indicating enhanced structural ductility and controlled failure. This study provides experimental evidence and theoretical support for the optimal design and engineering application of UHPC in flexural strengthening of RC beams.

## Introduction

 Global large-scale infrastructure construction has entered a stable phase, making the functional restoration and maintenance of existing structures a research focus in the engineering field. Currently, common strengthening methods include the following: The section enlargement method involves increasing the cross-sectional area or reinforcement ratio on the external surface of the existing member. Composite action between the original structure and the added layer enhances the overall flexural capacity and stiffness^1–3^. This method is simple and cost-effective, widely used in urban building strengthening projects. However, conventional concrete (C30-C50) has low tensile strength, often requiring thicker strengthening layers, which significantly increases the self-weight^4^.The steel plate/carbon fiber reinforced polymer (CFRP) bonding method uses epoxy resin adhesives to externally bond steel or CFRP plates. Composite action improves the overall load-bearing capacity^5–8^. The externally bonded plate increases the effective reinforcement ratio and protects the internal concrete, but its durability in corrosive environments remains a concern. The external prestressing method involves applying external prestressing tendons along the sides or bottom of the beam, transforming the reinforced concrete member into a partially prestressed concrete element. This enhances both load-bearing capacity and crack resistance^9,10^. However, exposed anchorage devices and prestressing strands are susceptible to durability issues due to environmental conditions.

Compared to traditional strengthening materials, UHPC offers significant advantages: (1) Excellent tensile strength (≥ 6 MPa), enabling thin strengthening layers with minimal weight increase; (2) Superior fluidity and cohesion, ensuring high bond strength with existing concrete structures and coordinated deformation; (3) Dense microstructure, providing outstanding durability.Owing to these benefits, UHPC has become a research hotspot in flexural strengthening^11–15^. Lampropoulos et al.^16^, Deng Zongcaiet et al.^17^, and Habel et al.^18^ experimentally confirmed that UHPC enhances the load-bearing capacity and ductility of existing RC beams, demonstrating its technical feasibility. Safdar et al.^19^ investigated the mechanical performance of UHPC-strengthened RC beams through experiments and numerical simulations, showing that the strengthening layer effectively improves beam stiffness and suppresses crack development. Zhu et al.^20^ and Zhang Yang et al.^21^ explored the influence of parameters such as the initial damage degree of RC beams, steel fiber orientation, and curing conditions on flexural performance, revealing that existing damage states and UHPC curing conditions affect the final strengthening effectiveness. Regarding the bond behavior at the UHPC-to-concrete interface, Sun Hangxing et al.^22^ noted that roughening treatment (roughness ≥ 3 mm) combined with steel bar planting can increase interfacial shear strength by over 40%, but shear key arrangement must be designed to avoid stress concentration. Xia Junrun’s^23^ cyclic loading tests indicated that an interface with planted steel bars retained 80% of its initial strength after 1 million cycles, whereas a conventionally roughened interface degraded to 60%. Thus, the combination of planted steel bars and UHPC grouting is recommended for applications under dynamic loads.

Current research on UHPC-strengthened RC beams primarily relies on the section enlargement method, replacing ordinary concrete with UHPC and using experimental data as the main design basis. Existing studies mostly employ traditional resistance strain gauges and crack observation instruments to measure key point strains and crack patterns, but full-field strain and deflection data are difficult to obtain. Numerical analyses often compare characteristic points such as cracking and ultimate loads with experimental results, but simulations of the entire load-displacement curve remain inaccurate. Given the limitations of existing experimental techniques and the inaccuracy of numerical analyses, this study starts from the material characteristics of UHPC. Uniaxial tension and compression tests were conducted to establish the constitutive model of UHPC, and the bond-slip behavior at the interface between new and old concrete was considered. A refined finite element model of UHPC-strengthened RC beams was developed to systematically analyze the entire flexural process. The accuracy of the numerical model was verified experimentally, and digital image correlation (DIC) technology was used to reveal the internal mechanisms of flexural capacity enhancement, stiffness improvement, and crack evolution in the strengthening system.

## Experimental program

### Ultra-high performance concrete material

The UHPC used for strengthening RC beams consisted of cement, silica fume, quartz sand, water, polycarboxylate-based superplasticizer, and steel fibers. The specific mix proportions are listed in Table 1. Portland cement (P.O 52.5 grade) conforming to the Chinese standard GB175-2018^24^ was used. Silica fume, a highly reactive mineral admixture, was added at one-third the mass of cement. The superplasticizer significantly influences the workability and mechanical properties of UHPC by effectively reducing the water-to-binder ratio. Based on multiple workability tests, the optimal dosage was determined as 0.8% of the total mass of cementitious materials (corresponding to 2.8 g in this mix). Steel fibers (13 mm in length) with a volume fraction of 1.5% were used to enhance the tensile and compressive strengths and crack resistance of UHPC^25,26^. The preparation process was as follows: Cement and silica fume were first added to a forced-action mixer and stirred clockwise for 2 min. Quartz sand was then added in the corresponding proportion, and mixing continued at the same speed for another 2 min. Steel fibers were uniformly dispersed into the mixer. Next, the superplasticizer and water were premixed into a solution and added to the mixer, followed by continuous mixing for 6 min. Afterward, the mixer’s rotation direction was reversed, and mixing continued for an additional 5 min to ensure homogeneity. The fresh UHPC mixture was cast into molds and placed on a vibrating table for 40–50 s to remove entrapped air. The specimens were then covered with plastic film and cured at 20 ± 3 °C and ≥ 95% relative humidity for 24 h. After demolding, the specimens were steam-cured at 95 °C for 72 h before testing.

**Table 1:** Mix proportions.

Material	Content
w/b ratio	0.2
Cement (g)	920
Silica fume (g)	276
Coarse sand (g)	810
Fine sand (g)	202
Steel fibers (g)	117.75 (1.5%)
Superplasticizer (g)	2.8

### Mechanical properties of UHPC

**Fig. 1:**
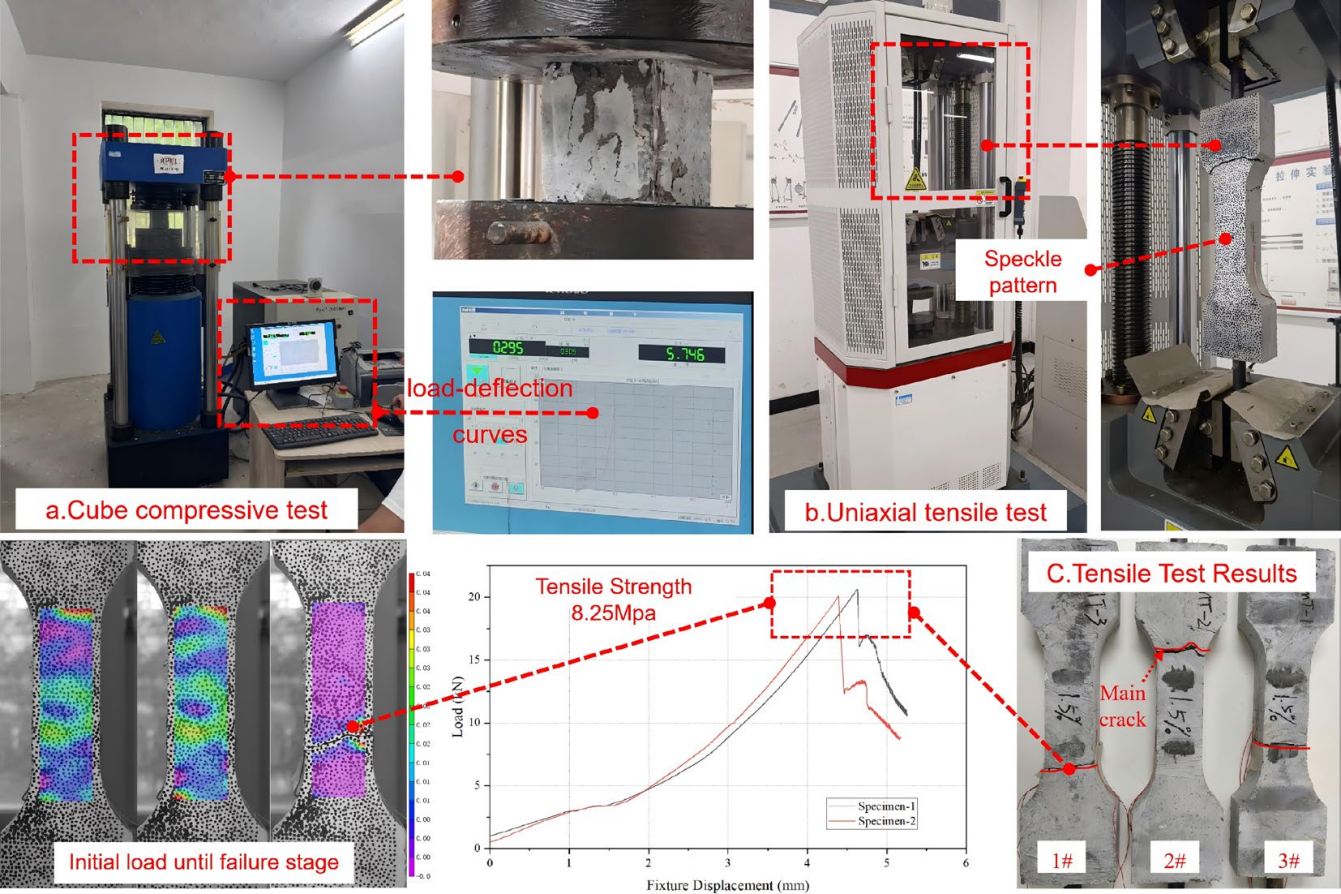
Mechanical properties test of UHPC.

The enhancement effect of the UHPC strengthening layer is directly related to its mechanical properties, which are also critical for subsequent finite element analysis. This study determined the mechanical properties of UHPC and related materials through experiments, as summarized in Table 2. To obtain the compressive and tensile behaviors, cube specimens (100 mm × 100 mm × 100 mm) and dumbbell-shaped specimens were prepared. Compressive tests were conducted on a 300-t hydraulic servo universal testing machine, as shown in Fig. 1a. In the UHPC-RC strengthening system, the strengthening layer primarily bears tensile stress, making its tensile performance crucial. Test methods for concrete tensile performance include direct tension, flexural tension, and splitting tension tests, which are not yet standardized internationally. Direct tension tests provide the true tensile strength and constitutive relationship but are complex and require specialized fixtures. The other two are indirect methods that are simpler but cannot directly provide the tensile constitutive relationship^27^. Therefore, this study adopted direct tension tests using dumbbell-shaped specimens with a total length of 368 mm and a thickness of 50 mm^28–30^ (specimen shape and dimensions are shown in Fig. 1b). During testing, the specimens were fixed to a 100-t tension testing machine via pre-embedded bolts. The full-field strain on the specimen surface was non-contact captured using DIC technology (detailed in “Testing methods” section), and the load–displacement curve was obtained by combining load data from the testing machine, as shown in Fig. 1c.

**Table 2:** Mechanical properties of materials.

Materials	Cube compressive strength/MPa	Modulus of elasticity/MPa	Poisson’s ratio	Tensile strength/MPa
C20 concrete	20.1	25,600	0.2	2.01
UHPC	150.6	46,140	0.2	8.25
HRB400 rebars	–	205,000	0.3	404 (yield strength)

### Strengthened RC beam test program

**Fig. 2:**
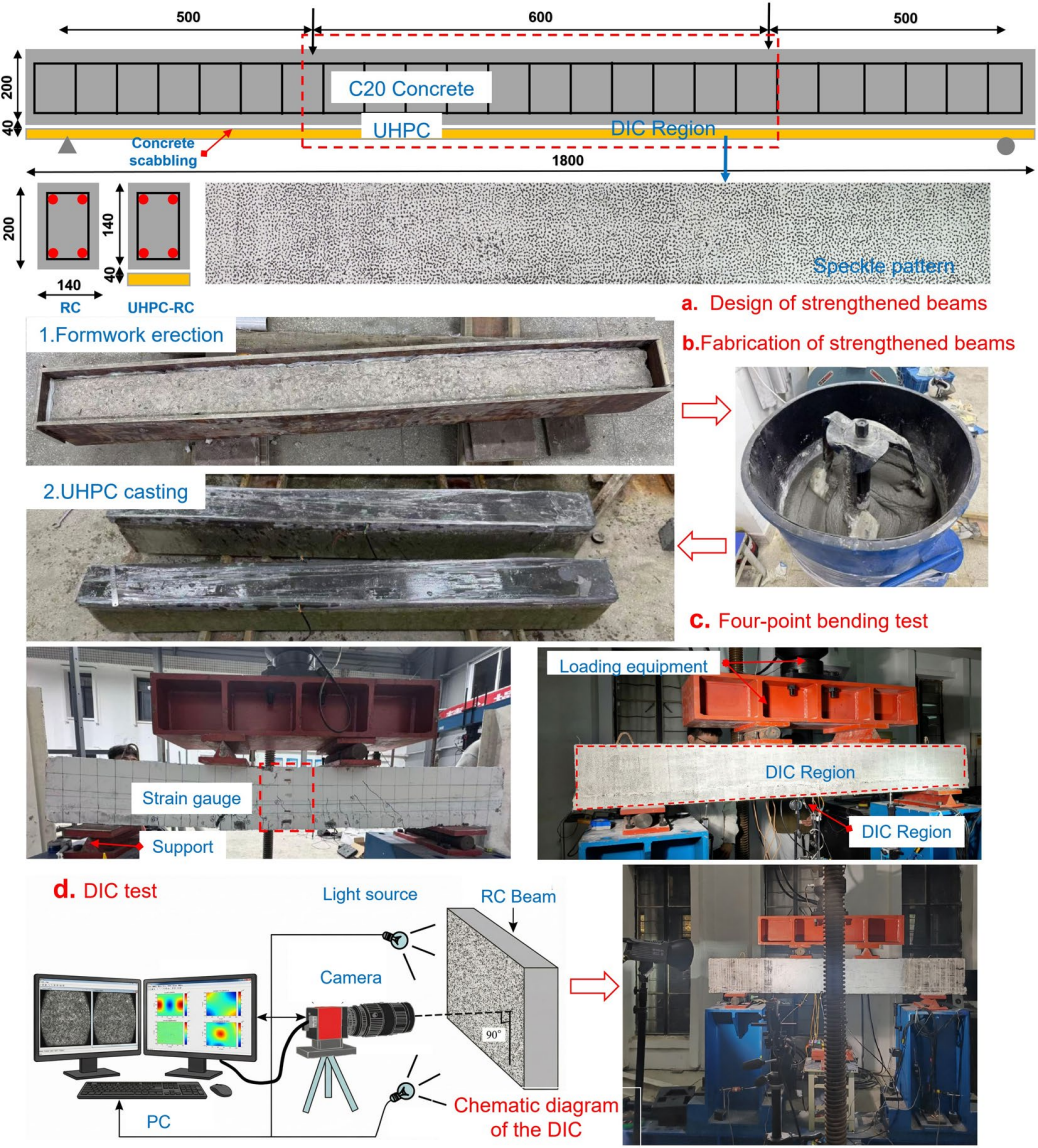
Fabrication and testing process of the test beam.

To analyze the mechanical behavior of the UHPC-RC beam system under four-point bending and validate the subsequent finite element model, three test beams were designed and fabricated: one unstrengthened RC control beam (designated CON) and two RC beams strengthened with a 40 mm thick UHPC layer (designated SY_40_ and SY_2 × 12_). Detailed parameters are listed in Table 3. The RC beam specimens had a cross-section of 140 mm (width) × 200 mm (height) and a clear span of 1800 mm. The UHPC strengthening layer dimensions were 140 mm (width) × 40 mm (thickness) × 1800 mm (length). The concrete grade for the beam was designed as C20. The longitudinal tensile reinforcement consisted of two 12 mm diameter HRB400 bars (measured yield strength 404 MPa, as shown in Table 2), and stirrups were 8 mm diameter HPB300 bars spaced at 50 mm. To ensure effective bonding between the UHPC strengthening layer and the original C20 concrete beam bottom, the bonding surface was standardized roughening treatment: the roughening depth was controlled at 3–5 mm to form a uniformly rough surface (average roughness Ra ≥ 0.8 mm); after treatment, surface laitance and debris were removed, exposing coarse aggregate area ≥ 60%, and the interface was kept clean and dry before UHPC casting. The UHPC material preparation and curing process for the strengthened beams were the same as in “Ultra-high performance concrete material” section. During the four-point bending test, full-field non-contact measurement of the displacement and strain fields in the constant-moment region of the mid-span was performed using DIC technology; simultaneously, resistance strain gauges and surface strain meters were used for data verification. Figure 2a and b show the detailed design and actual specimens of the test beams; Fig. 2c illustrates the test loading setup; Fig. 2d shows the arrangement of the data acquisition system.

**Table 3:** Parameters of test beams.

No.	Beam composition	Beam ID	Original concrete grade	Reinforcement parameters
Group 1	RC beam	CON	C20	Control group
Group 2	Strengthened beam: 40 mm thick UHPC layer	SY_40_	C20	Test group
Group 3	Strengthened beam: 40 mm UHPC layer + 2 Φ12	SY_2 × 12_	C20	Test group

### Testing methods

The UHPC cube compressive test was conducted according to the “Basic Performance and Test Methods of Ultra-High Performance Concrete”^30^. Stress-controlled loading was applied at a rate of 1.0 MPa/s until specimen failure. During initial loading, fine audible cracks appeared, and microcracks gradually developed. As the load increased, cracks extended and eventually formed penetrating main cracks, accompanied by a clear brittle failure sound, indicating loss of bearing capacity. The average compressive strength of UHPC cube specimens was 150.6 MPa. The UHPC direct tension test followed the same standard, using dumbbell-shaped specimens loaded at a displacement rate of 0.5 mm/min on a tension testing machine. To capture the full-field strain on the specimen surface, speckle patterns were sprayed on the central region (100 mm × 50 mm) before testing, and images were acquired at 1 frame/s using a 25-megapixel digital camera. Combined with DIC technology, the strain and displacement responses during the entire tensile process were analyzed. The average tensile strength of UHPC was 8.25 MPa. In the four-point bending test of strengthened RC beams, the load was applied via a hydraulic actuator and reaction frame system. Speckle patterns were sprayed on one side of the beam, and the DIC system captured the full-field displacement and strain evolution in the pure bending segment (600 mm × 150 mm) at the mid-span at a frequency of 1 frame/3 s. Resistance strain gauges and displacement meters were symmetrically arranged on the other side for data verification. The setup of the loading and data acquisition systems is shown in Fig. 2c and d.

## Experimental results

### Flexural behavior of test beams

**Fig. 3:**
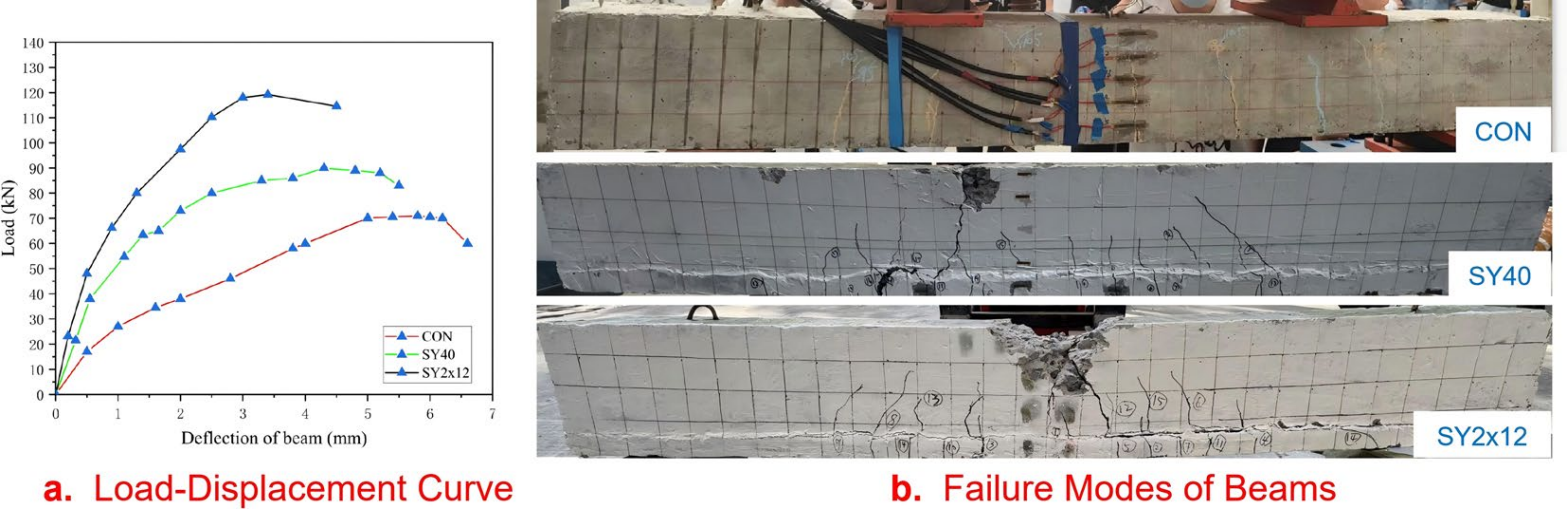
Load-displacement curve and failure diagram of test beam.

Figure 3a shows the load–displacement curves of the test beams. The curves of the two UHPC-strengthened beams (SY_40_ and SY_2 × 12_) are significantly higher, indicating that the UHPC strengthening layer markedly enhances the flexural capacity of RC beams. The curves of the strengthened beams show distinct inflection points during the ascending branch, reflecting differential strengthening effects at various loading stages. The experimental data are clear and reliable. In this study, the cracking load was defined as the point where the curvature of the ascending branch significantly changes, and the ultimate load was defined as the peak point. Compared to the unstrengthened control beam (CON): The beam strengthened with 40 mm UHPC (SY_40_) showed a 40.7% increase in cracking load and a 26.8% increase in ultimate load. The beam strengthened with UHPC and 2 × 12 mm bars (SY_2 × 12_) exhibited a 145.2% increase in cracking load and a 67.9% increase in ultimate load. In terms of flexural stiffness (evaluated at the displacement corresponding to the ultimate load), the stiffness of SY_40_ increased by 25.9%, and that of SY_2 × 12_ increased by 41.4% compared to the CON beam.

### Analysis of failure modes

The final failure modes of the strengthened and control beams are shown in Fig. 3b. Cracks were mainly concentrated in the mid-span constant-moment region, developing vertically from the beam bottom to the top with relatively large widths. A few diagonal cracks appeared outside the loading points, exhibiting typical flexural failure characteristics, consistent with experimental expectations.

**Fig. 4:**
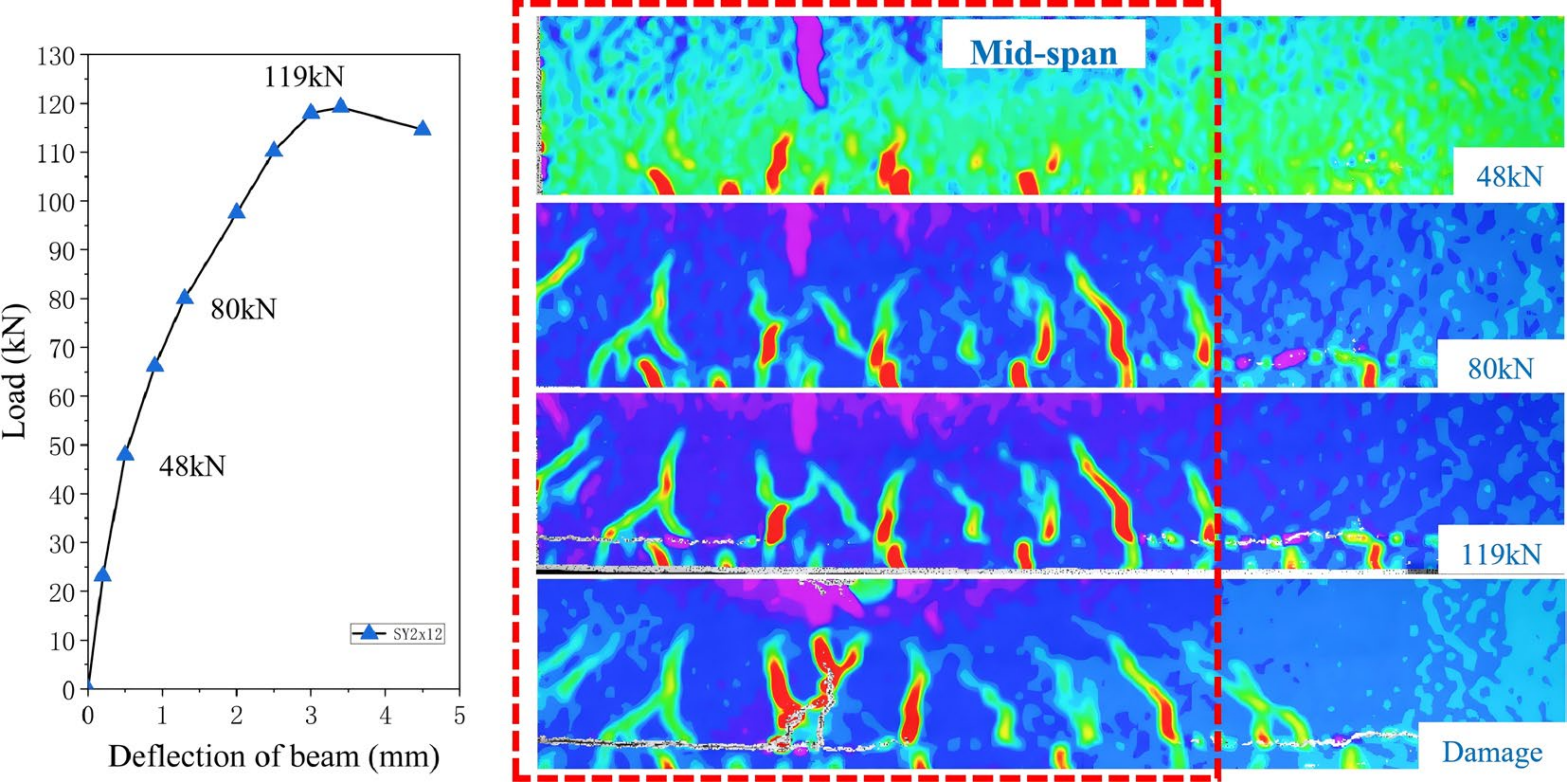
DIC test results.

DIC technology was used to measure the strain distribution of the test beams, as shown in Fig. 4: The unstrengthened control beam (CON) primarily developed vertical cracks, which extended above the neutral axis at failure.The UHPC-strengthened beams (SY_40_ and SY_2 × 12_) showed similar crack propagation patterns: cracks first initiated in the UHPC layer, then extended to the interface between new and old concrete, where horizontal cracking occurred, followed by upward propagation into the RC beam, ultimately leading to beam failure. To further analyze the failure characteristics during the entire loading process, the SY_2 × 12_ test beam was taken as an example. DIC technology monitored its crack development process, strain field distribution, and failure mode, as shown in Fig. 4: Initial cracking stage (load ≈ 48 kN): High-strain zones appeared within the 40 mm thick strengthening layer at the beam bottom, indicating crack initiation, mainly forming four initial cracks. The mid-span region at the beam top exhibited high compressive strain zones corresponding to the cracking positions. The load–displacement curve showed an inflection point but remained approximately linear. Crack propagation stage (load ≈ 80 kN): The high-strain zones at the beam bottom expanded from four to seven cracks, which propagated upward and crossed the interface, causing cracking in the C20 concrete. The DIC strain field revealed that some C20 concrete cracks were direct continuations of UHPC layer cracks; others were not aligned with the lower cracks, formed by the merging of multiple UHPC cracks and subsequent upward propagation. Crack paths shifted horizontally after crossing the interface, related to interfacial bond performance: high bond stiffness ensured coordinated deformation, while local stress exceeding bond strength caused transverse slip and crack path deviation. During this stage, stresses in the UHPC layer and internal reinforcement increased significantly. Ultimate state (load ≈ 119 kN): The structure reached its maximum bearing capacity, and the tensile reinforcement yielded. The number of cracks stabilized, but widths increased further, extending into the compression zone. Local horizontal cracks appeared at the interface, indicating partial debonding without overall delamination. The concrete in the compression zone was under high stress, approaching failure. Failure stage: With further load increase, two intersecting main cracks formed in the mid-span region, the bottom reinforcement yielded, and the top concrete crushed, leading to rapid displacement increase until final failure. Notably, only local horizontal cracking occurred between the new and old concrete, without debonding or overall separation, demonstrating that roughening treatment and cast-in-place UHPC provided sufficient interfacial bond strength to prevent bond failure during flexural loading.

## Finite element analysis

### Finite element model

**Fig. 5:**
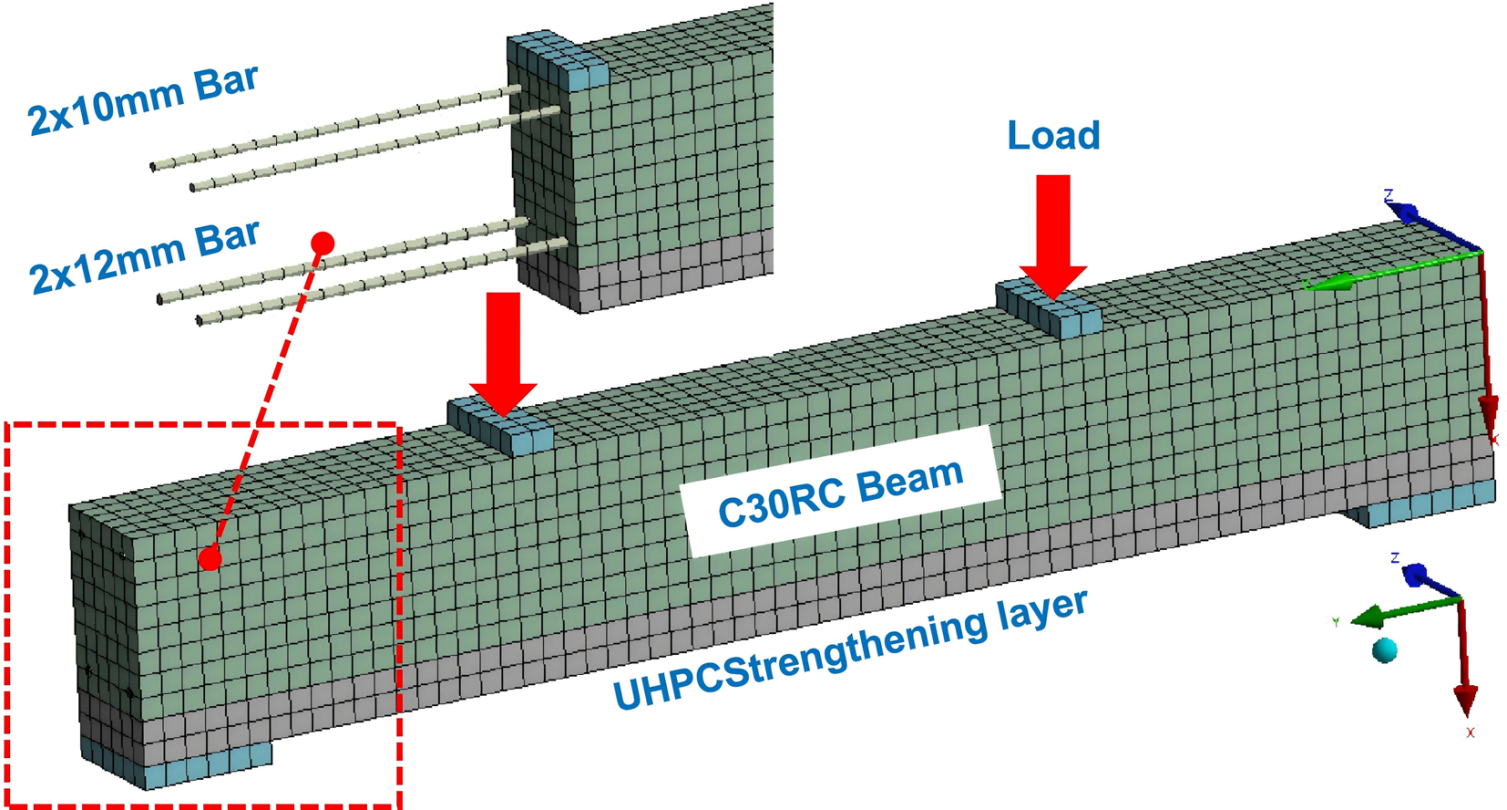
Finite element simulation model.

To simulate the mechanical behavior of UHPC-strengthened RC beams under four-point bending, a finite element model was established and validated against experimental beams with identical parameters. The model parameters are listed in Table 4, where specimens UR_40_ and UR_2 × 12_ correspond to the test beams in Chap. 3, and other specimens were used for parametric analysis, with variables including UHPC strengthening layer thickness, compressive strength (120 MPa, 150 MPa, 180 MPa), and reinforcement ratio in the UHPC layer. Finite element analysis of reinforced concrete structures is commonly performed ANSYS Workbench (WB). In WB, reinforced concrete modeling mainly employs smeared and discrete approaches. The smeared model uses Solid65 elements with reinforcement ratios defined via real constants, typically using MISO or similar models to describe elastic and hardening stages but unable to simulate the softening branch (descending part of the constitutive curve), making it unsuitable for plastic damage analysis of RC beams. Therefore, this study proposes a discrete modeling method based on WB: concrete is modeled with Solid185/186 elements, using the DPC (Drucker-Prager Concrete) and MWC (Menetrey-Willam Concrete) constitutive models to describe the stress-strain behavior, and linear/power/exponential functions combined to simulate the softening stage (detailed in “Material constitutive relationship model” section). Reinforcement can be modeled with Link180 beam elements or Reinf264/265 reinforcement elements. Boundary conditions and loading methods significantly affect convergence and accuracy. To avoid stress concentration, rigid steel plates were set at the bottom supports and mid-span loading points (Fig. 5). The left support constrained translations in x, y, and z directions; the right support constrained x and z translations. Displacement-controlled loading was applied to the mid-span rigid plate. The interface between the UHPC strengthening layer and the existing concrete beam bottom was simulated using surface-to-surface contact.

**Table 4:** Parameters of simulated beams.

No.	Test beam composition	Beam ID	Parameters	Remarks
1	Control Beam	RC	Control Group	RC beam
2	Simulated beam: Effect of UHPC Thickness	UR_40−60_	Thickness: 40, 50, 60 mm	UHPC strength: 150 MPa
3	Simulated beam: Effect of Strengthening Material	UR_C30_,UR_40_	Material strength	UHPC strength: 150 MPa
4	Simulated beam: Effect of UHPC Strength Grade	UR_120_,UR_150,_UR_180_	Different UHPC strength grades	Strengths: 120, 150, 180 MPa
5	Simulated beam: Effect of Reinforcement Ratio	UR_2 × 6_,UR_2 × 12_	Different Reinforcement Ratio	2 bars of 6 mm; 2 bars of 12 mm

### Material constitutive relationship model

The selection of material constitutive models directly affects the accuracy of calculation results. Based on UHPC direct tension test data, the tensile constitutive model of UHPC was established using the Menetrey-Willam Concrete (MWC) criterion. The compressive constitutive model of UHPC, the tensile and compressive constitutive models of C30 concrete, and the tensile constitutive model of HRB400 reinforcement were adopted from relevant current codes.

1. Constitutive model of C30 concrete​

The uniaxial tensile stress-strain relationship of concrete is defined as follows^31^:

1 $$\sigma\:=(1-{d}_{t}){E}_{c}\epsilon\:$$

2 $$d_{t} = \left\{ {\begin{array}{*{20}l} {1 - \rho _{t} [1.2 - 0.2x^{5} ]} \hfill & {x \le 1} \hfill \\ {1 - \frac{{\rho _{t} }}{{\alpha _{t} (x - 1)^{{1.7}} + x}}} \hfill & {x > 1} \hfill \\ \end{array} } \right.$$

3 $$x = \frac{\varepsilon }{{\varepsilon _{{t,r}} }}$$

4 $$\rho _{t} = \frac{{f_{{t,r}} }}{{E_{c} \varepsilon _{{t,r}} }}$$

The uniaxial compressive stress-strain relationship is defined as follows^31^:

5 $$\sigma = (1 - d_{c} )E_{c} \varepsilon$$

6 $$d_{c} = \left\{ {\begin{array}{*{20}l} {1 - \frac{{\rho _{c} n}}{{n - 1 + x^{n} }}} \hfill & {x \le 1} \hfill \\ {1 - \frac{{\rho _{t} }}{{\alpha _{c} (x - 1)^{2} + x}}} \hfill & {x > 1} \hfill \\ \end{array} } \right.$$

7 $$\rho _{n} = \frac{{f_{{c,r}} }}{{E_{c} \varepsilon _{{c,r}} }}$$

8 $$n = \frac{{E_{c} \varepsilon _{{c,r}} }}{{E_{c} \varepsilon _{{c,r}} - f_{{c,r}} }}$$

9 $$x = \frac{\varepsilon }{{\varepsilon _{{c,r}} }}$$

where: *E*_*c*_—Represents the elastic modulus of concrete, *d*_*t*_, *d*_*c*_—Denote the damage evolution parameters of concrete under uniaxial tension and compression, respectively. *α*_*t*_, *α*_*c*_—The parameters defining the descending branch of the constitutive curve. *f*_*t,r*_, *f*_*c,r*_—Correspond to the characteristic values of uniaxial tensile and compressive strength, respectively. $\varepsilon _{{t,r}} ,\varepsilon _{{c,r}}$—Indicate the peak strains corresponding to the characteristic uniaxial tensile and compressive strengths, respectively.

The material parameters for C30-grade concrete are specified as follows: *E*_*C*_ = 32500 MPa, ${\mathrm{f}}_{\mathrm{t,r}}$ = 1.43 MPa, ${\mathrm{f}}_{\mathrm{c,r}}$ = 14.3 MPa, $\varepsilon _{{t,r}} = 81$ × 10^−6^, $\varepsilon _{{c,r}} =$ 1640 × 10^−6^^31^.

**Fig. 6:**
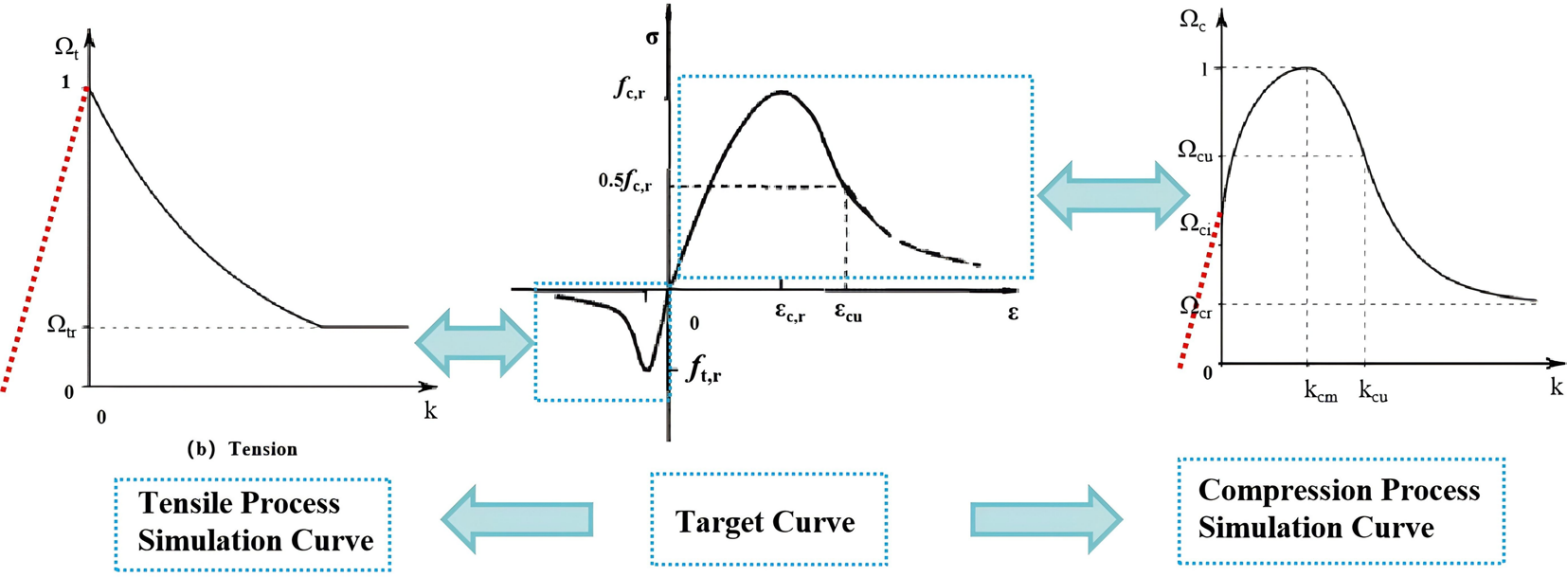
Concrete constitutive curve.

The MWC criterion adopted in this study employs the following parameters: uniaxial compressive strength *R*_c_ = *f*_*c, r*_ = 14.3 MPa, uniaxial tensile strength *R*_*t*_ = ${\mathrm{f}}_{\mathrm{t,r}}$ = 1.43 MPa, biaxial compressive strength *R*_*b*_ = 17.16 MPa, and a dilation angle of 30°. After the material enters the hardening and softening stages, its constitutive behavior is described by the yield functions *Ω*_*c*_(for compression) and *Ω*_*t*_ (for tension). The stress-strain relationships of the material under uniaxial compression and tension are illustrated in Fig. 6.

In the figure, the red dashed line represents the elastic stage of the material, whose constitutive relationship is considered linear. After entering the hardening and softening stages, the simulated curve exhibits nonlinear changes, indicating that the material has entered the plastic stage. This behavior is described by the yield function *Ω*, where the horizontal axis represents the strain *k*, and the vertical axis corresponds to the normalized stress ratios *Ω*_*c*_ and *Ω*_*t*_ for the compressive and tensile directions, respectively. *Ω* is defined as the ratio of the current stress to the peak stress; therefore, the maximum values of *Ω*_*c*_ and *Ω*_*t*_ are 1. The relevant parameters for C30 concrete are defined as follows: the plastic strain at the peak point *k*_*cm*_ = *ε*_*c, r*_ = 1640 × 10^− 6^; the ultimate compressive plastic strain *k*_*c*r_=0.01; the relative stress at the onset of hardening *Ω*_*ci*_ = 0.4; and the residual relative compressive stress *Ω*_*cr*_ = 0.2. The piecewise expression for the yield function *Ω*_*c*_ is as follows: when *k* < *k*_*cm*_, *Ω*_*c*_ is calculated using Eq. (10); when *k*_*cm*_<*k*<*k*_*cu*_, *Ω*_*c*_ is calculated using Eq. (11); and when *k* > *k*_*cu*_, it is calculated using Eq. (12).

10 $${\varOmega\:}_{c}={\varOmega\:}_{ci}+(1-{\varOmega\:}_{ci})\sqrt{2\frac{k}{kcm}-\frac{{k}^{2}}{{kcm}^{2}cm}}$$

11 $${\varOmega\:}_{c}=1-(1-{\varOmega\:}_{cu}){\left(\frac{k-kcm}{kcu-kcm}\right)}^{2}$$

12 $${\varOmega\:}_{c}={\varOmega\:}_{cr}+({\varOmega\:}_{cu}-{\varOmega\:}_{cr})exp\left(2\frac{{\varOmega\:}_{cu}-1}{kcu-kcm}\frac{k-kcu}{{\varOmega\:}_{cu}-{\varOmega\:}_{cr}}\right)$$

Similarly, the tensile constitutive model of concrete adopts the MWC criterion. The tensile process consists of an elastic stage and a softening stage. The softening stage employs a linear softening model, with the main parameters being: the ultimate tensile plastic strain *k*_*tr*_=0.01, and the residual relative tensile stress *Ω*_*t*r_ = 0.1.

(2) Constitutive model of UHPC

The tensile and compressive constitutive models of UHPC are also based on the MWC criterion. According to the experimental results, the compressive stress-strain relationship curve is nonlinear and described by Eqs. (10)–(12), while the tensile stress-strain relationship curve is linear. The main parameters are as follows: UHPC uniaxial compressive strength *R*_*c*_*=f*_*c, r*_*=*150.6 MPa, uniaxial tensile strength *R*_*t*_*=f*_*t, r*_*=*8.25 MPa, biaxial compressive strength *R*_*b*_ = 180.7 MPa, dilation angle = 30°, and elastic modulus = 46,140 MPa. The plastic strain at the peak point *K*_*cm*_  =  *ε*_*c, r*_ = 1870 × 10^− 6^; the ultimate compressive plastic strain *k*_*cr*_ = 0.01; the relative stress at the onset of hardening *Ω*_*ci*_ = 0.65, and the residual relative compressive stress *Ω*_*cr*_ = 0.25; the ultimate tensile plastic strain *k*_*tr*_ = 0.01; and the residual relative tensile stress *Ω*_*tr*_ = 0.48.

**Fig. 7:**
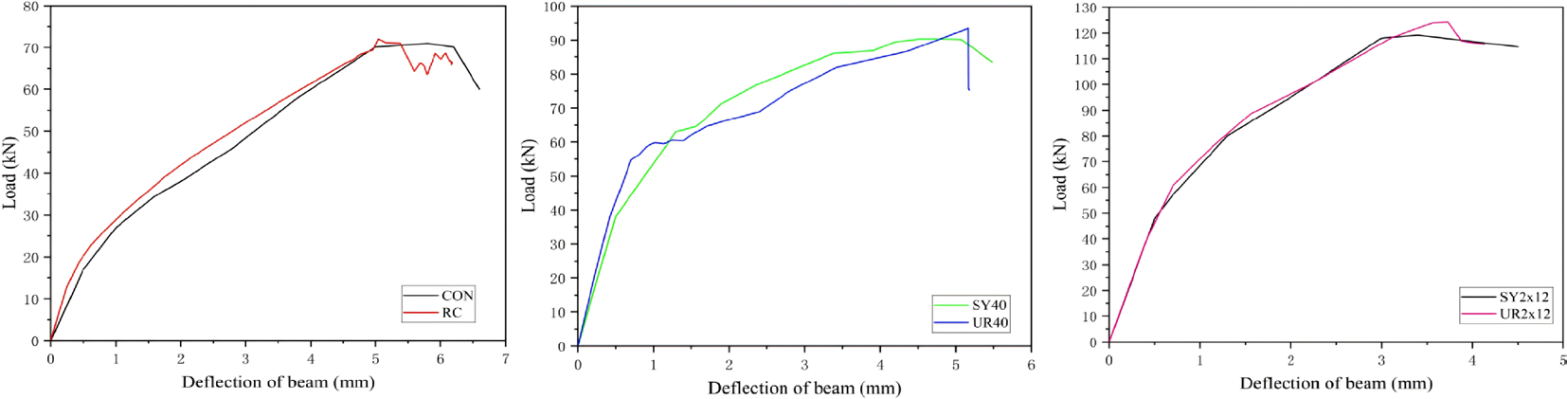
Comparison of simulation and experimental results.

(3) The reinforcement adopts a bilinear ideal elastoplastic constitutive curve, with parameters based on experimental results: yield strength of 404 MPa and elastic modulus of 205 GPa.
(4) UHPC-RC interface bond model.

- The bond behavior at the interface between new and old concrete is a critical aspect of finite element simulation. Existing interface models primarily include two types: those that only provide the ultimate bond strength, and constitutive models capable of simulating the entire failure process of the interface. This study adopts the latter approach, utilizing the bond-slip model proposed by Neubauer and Rostásy^32,33^. The relationship is illustrated in Fig. 7. In this model, the interfacial stress increases linearly with slip until reaching the ultimate bond strength $\tau$_u_. After attaining $\tau$_*u*_, the bond strength drops abruptly to zero. Based on experimental results from the literature and comparison with simulated and experimental load-displacement curves^34,35^, the interface parameters are determined as: $\tau\:$_*u*_=3.07 MPa, *S*_*u*_=0.294 mm, and *K* = 10.4 MPa/mm. Using this set of parameters, the simulation results show good agreement with experimental data. In the numerical model, the interface bond behavior is simulated using COMBIN39 nonlinear spring elements, and the bond-slip relationship is characterized by its force-displacement (F-D) curve. Experimental results indicate that interfaces treated with roughening show no debonding of the UHPC strengthening layer during loading, demonstrating that this treatment method provides reliable interfacial bond performance. This confirms the applicability and safety of the method in practical engineering.
- The model is described using Expression (13):

13 $$\tau\:=\left\{\begin{array}{ll}Ks&\:\hspace{1em}0\le\:s<{s}_{u}\\\:0&\:\hspace{1em}{s}_{u}\le\:s\end{array}\right.\:$$

where: *K*—Interfacial bond stiffness (slope of the linear ascending branch), MPa/mm. *s*—Interfacial slip amount, mm. *S*_*u*_—Slip corresponding to the bond strength (peak slip amount), mm. *τ*—Interfacial bond stress, MPa. *τ*_*u*_—Ultimate bond strength, MPa.

### Calculation results and discussion

#### Comparison of simulation and experimental results

To improve computational convergence, multiple rounds of debugging were performed on the load steps in the finite element analysis. The final settings were determined as follows: the initial increment was set to 10, the maximum allowable increments were set to 3000–5000, and a direct integration solver (Static, General) was selected. Meanwhile, to balance computational accuracy and efficiency, the global mesh size of the model was set to 20 mm. During the calculation process, the judgment of whether the structure reached its ultimate state was based on the following criteria: (1) The stress in the tensile reinforcement reached its specified yield strength (404 MPa); (2) Concrete or UHPC elements exhibited failure phenomena such as severe distortion, crushing, or tensile fracture. If the calculation results did not meet any of the above criteria, the increment steps were adjusted for recalculation. A comparison between the simulation results and experimental data is shown in Fig. 7; Table 5. The simulation error for the cracking load ranged from 0.49 to 6.25%, and the error for the ultimate load ranged from 1.45 to 4.20%. The finite element model effectively reproduced the mechanical characteristics of the strengthened beams: the ultimate load and mid-span deflection closely matched the experimental values, and the model accurately reflected the stiffness variations and strength development in all stages, including the elastic stage, hardening stage, and softening stage. The above results indicate that the finite element model established in this study demonstrates high computational accuracy.

**Table 5:** Comparison of calculation results.

Beam ID	Cracking load/kN	Ultimate load/kN	Maximum displacement/mm
CON	27.00	71.00	5.80
RC	26.08	72.03	5.04
SY_40_	38.00	90.00	4.30
UR_40_	37.81	93.44	4.86
SY_2 × 12_	66.2	119.20	3.40
UR_2 × 12_	60.54	124.21	3.73

#### Parametric analysis of finite element simulation

1. Flexural behavior

**Fig. 8:**
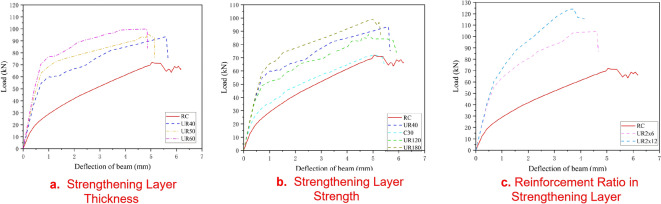
Load displacement curve.

**Fig. 9:**
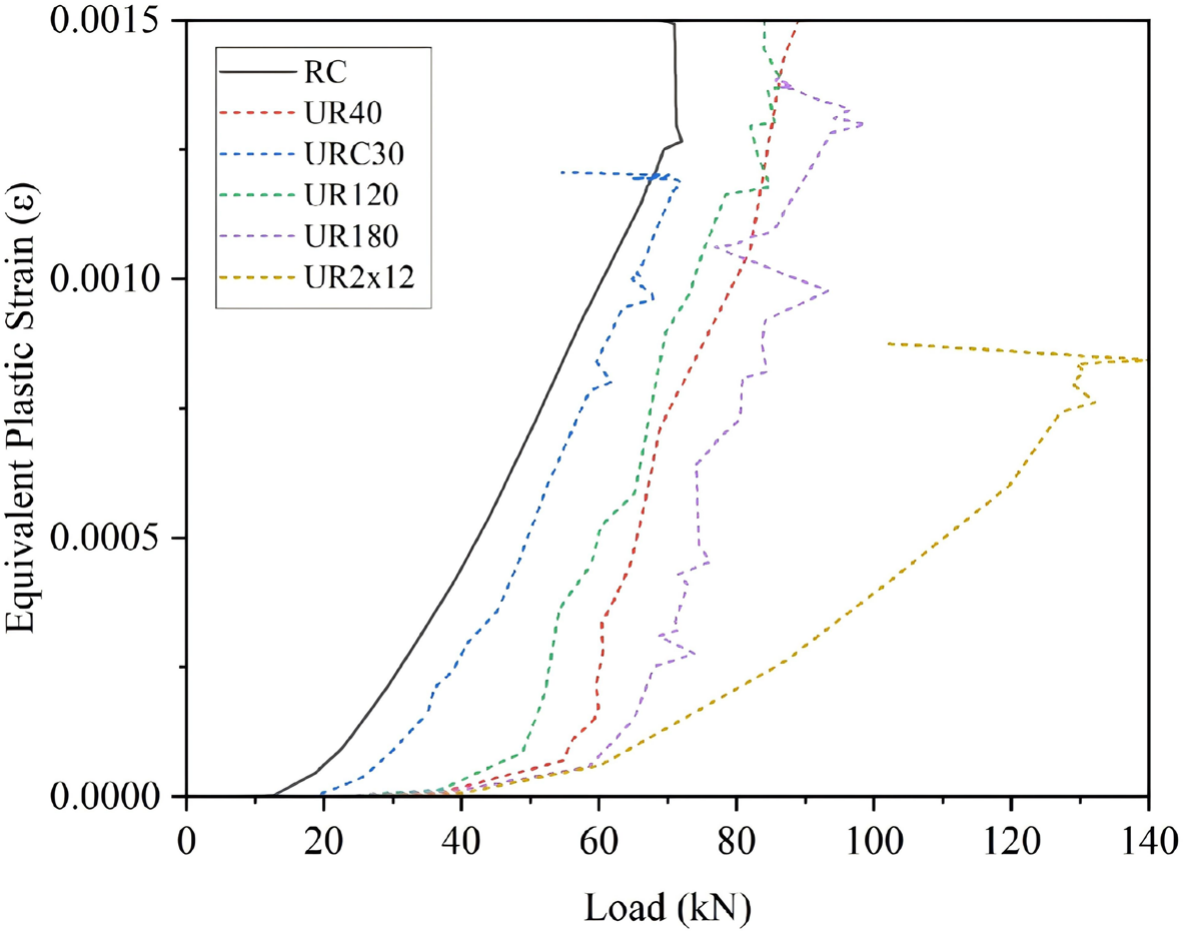
Equivalent plastic strain diagram of RC beam bottom.

Figure 9 illustrates the load-displacement curves of strengthened beams under various parameter conditions. (1) Effect of strengthening layer thickness: In the case of unreinforced UHPC, as the thickness of the UHPC strengthening layer increases, the cracking load, ultimate load, and stiffness of the beam increase accordingly. The cracking load improvement ranges from 45 to 84%, and the ultimate load improvement ranges from 29.7 to 38.4%, as shown in Fig. 8a. (2) Effect of strengthening material strength: When C30 concrete is used as the strengthening layer, the ultimate load of the test beam shows little improvement, while the cracking load increases by 16.1%. When UHPC material is used, as the strength increases (120 MPa, 150 MPa, and 180 MPa), the cracking load, ultimate load, and stiffness of the beam increase accordingly. The cracking load improvement ranges from 38.1 to 47.7%, and the ultimate load improvement ranges from 21.4 to 37.3%. The UHPC strengthening method exhibits better working performance compared to the traditional section enlargement method, as shown in Fig. 8b. (3) Effect of reinforcement in the UHPC layer: After considering reinforcement in the UHPC strengthening layer of the UHPC-RC composite system, the crack resistance increases by 114.1–132.1%, and the ultimate load increases by 45.1–72.4%. The UHPC-RC strengthening system with a certain proportion of reinforcement can significantly improve the load-bearing capacity and service performance of existing structures, as shown in Fig. 8c.

(2) Evolution law of plastic strain in the original beam

To reveal the cracking mechanism of UHPC-RC strengthened beams under load, this paper uses the equivalent plastic strain (PEEQ) to characterize the crack development pattern. The results are shown in Fig. 9. After the load exceeds the cracking load, the PEEQ of each beam begins to increase, indicating the initiation of cracks. The curves in the figure represent the average maximum equivalent plastic strain of the elements at the bottom of the unstrengthened RC beam (solid line) and each strengthened beam (dashed line). Compared with the unstrengthened RC beam, the equivalent plastic strain of the beam strengthened with C30 concrete (UR_C30_) under the same load decreases, indicating that crack development is somewhat suppressed, but the improvement is limited. In contrast, the equivalent plastic strain of the concrete at the bottom of the RC beam strengthened with UHPC (UR_120_, UR_150_, UR_180_) decreases significantly, indicating that the UHPC layer can effectively inhibit crack development. Before reaching the cracking load (strain approximately 57–85 µε), the curve rises slowly, and the rising slope is positively correlated with the material strength. At this stage, the equivalent plastic strain of UR_120_, UR_150_, and UR_180_ decreases by 44%, 79%, and 93%, respectively, compared to the unstrengthened beam. After exceeding the cracking load, cracks develop rapidly, and the rising slopes of the curves of the three UHPC-strengthened beams are basically the same, indicating that crack development at this stage is mainly controlled by the thickness of the strengthening layer and has little relationship with the material strength. The equivalent plastic strain of the UHPC-strengthened beam with reinforcement (UR_2 × 12_) remains at the lowest level throughout the loading process, and the curve rises gently after cracking, indicating that the crack development speed is slower, and the reinforcement in the UHPC layer has begun to bear tensile stress. Before the reinforcement yields, the crack width of this strengthened beam is always smaller than that of the unstrengthened beam. In summary, the UHPC layer with reinforcement can not only significantly improve the load-bearing capacity of the structure but also effectively control the initiation and propagation of cracks.

(3) Failure process

**Fig. 10:**
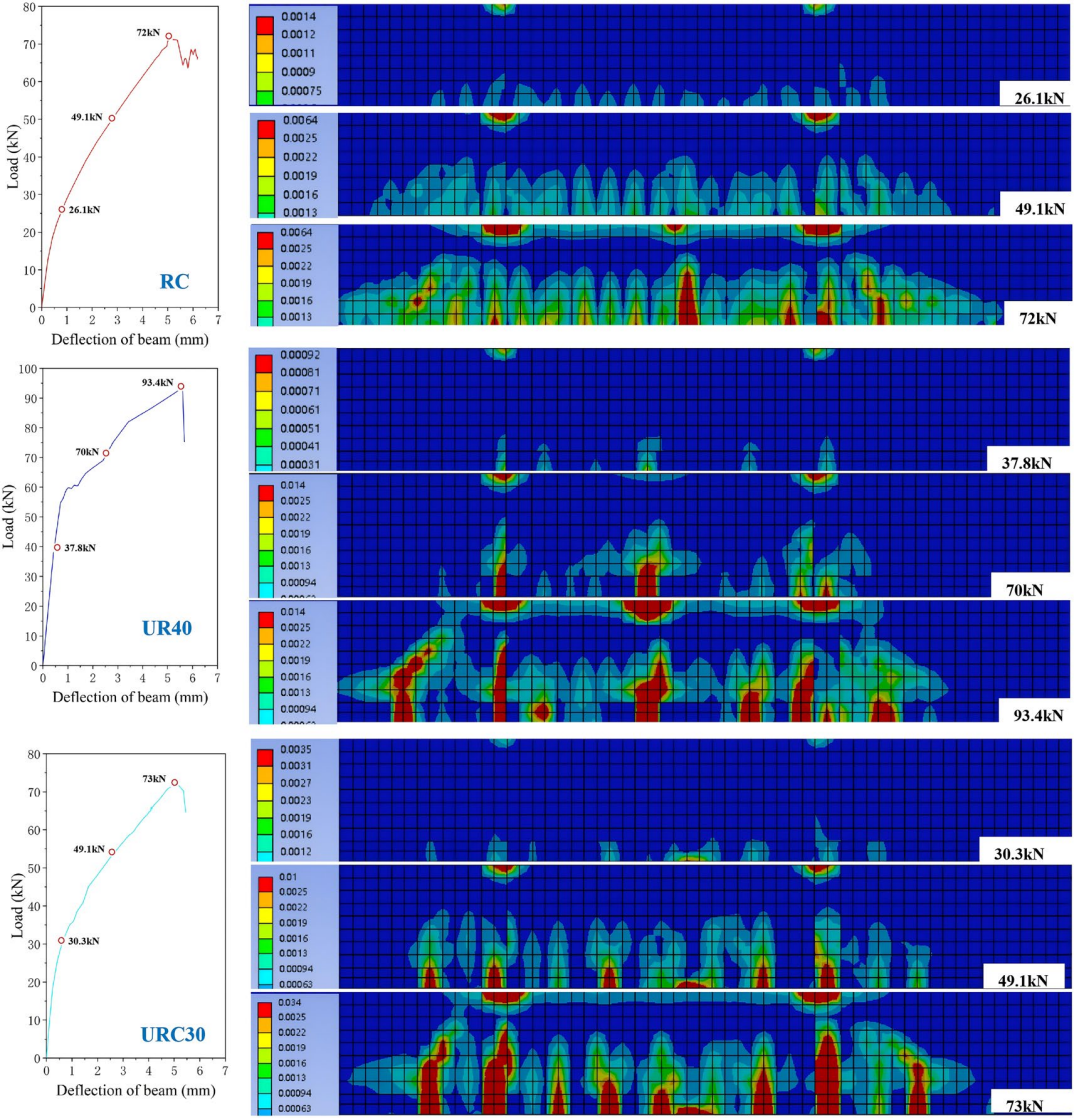

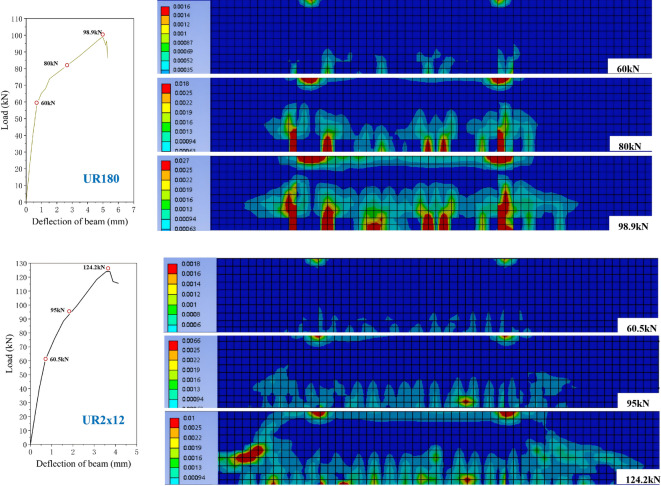
The process of beam failure.

Figure 10 shows the distribution of equivalent plastic strain in each strengthened beam from the hardening stage to the ultimate load stage. Combined with the load-displacement curve, the force process of the strengthened beam can be divided into three stages: (1) Elastic stage: The load-displacement relationship is basically linear; the concrete, reinforcement, and UHPC strengthening layer are well connected and deform coordinately. (2) Plastic hardening stage: The curve is still approximately linear but the slope decreases; the concrete enters the plastic stage, and minor slip occurs at the interface. (3) Softening stage: After the curve reaches the peak, the tensile reinforcement yields (stress reaches 404 MPa), the concrete in the tensile zone and the UHPC reach their tensile strength, the concrete in the compression zone is crushed, then the curve shows a brief decline, and finally the model fails due to distortion of local elements. The following analyzes the cracking characteristics of each beam based on the cloud diagrams of equivalent plastic strain of the simulated strengthened beams at each bending stage (taking the average load level of that stage):

UR_40_ strengthened beam: Compared with the control beam, the cracking load is significantly increased, and the crack distribution at the ultimate state is more concentrated, with increased spacing of main cracks. This benefits from the high tensile strength of UHPC (about 4–5 times that of ordinary concrete), which effectively inhibits the crack development in the RC beam. In the plastic hardening stage, a high plastic strain zone appears in the mid-span, and the strain distribution is sparser than that of the control beam, indicating more significant stress concentration, presenting the characteristic of “wide cracks and small number”. As the load increases, the number and width of main cracks in the tensile zone further increase, and the cracks between the RC beam and the strengthening layer at the mid-span show horizontal misalignment, which is consistent with experimental observations. The crushing range of the concrete in the compression zone is larger than that of the control beam, and the load-displacement curve drops steeply, indicating that the failure of the UR_40_ beam is sudden, the steel fibers inside do not fully exert their crack resistance effect, and the stress concentration at the beam bottom leads to premature failure of the structure.

UR_C30_ strengthened beam: Due to the increase in section height of 40 mm, its cracking load and initial stiffness are significantly improved, but the improvement in bearing capacity is limited by the tensile strength of C30 concrete.

UR_180_ strengthened beam: Due to the increased strength of UHPC, the cracking load is further increased. The crack development law during the plastic hardening and softening stages is basically similar to that of the UR40 beam, with sparse distribution of high-strain zones, also showing the characteristic of “wide and few” cracks. The load-displacement curve also drops sharply, indicating that although the increase in material strength improves the cracking and ultimate loads, the problem of stress concentration at the beam bottom has not been alleviated, and the material performance has not been fully utilized.

UR_2 × 12_ strengthened beam: After configuring two 12 mm diameter steel bars in the UHPC layer, the cracking load and ultimate load are further improved. The crack distribution is uniform and develops slowly, and the crack width and length are smaller than those of other beams. The plastic strain distribution of the RC beam is dense, indicating that the material advantages of UHPC are fully utilized, which is in sharp contrast to beams such as UR40 and UR180. In the softening stage, the crack width is still much smaller than that of other beams, and the load-displacement curve drops gently and shows a step-like shape, indicating that the UHPC layer and its internal reinforcement jointly bear the load, significantly improving the flexural bearing capacity and ductility of the RC beam.

The UHPC layer with a certain amount of reinforcement can not only effectively improve the bearing capacity but also improve the crack distribution, delay the failure process, and show better strengthening effect.

## Conclusions

Based on material tests and strengthening experiments, this study established a multi-parameter finite element simulation model to evaluate the flexural behavior of the UHPC-RC strengthening system. The main conclusions are as follows:

1. The plastic constitutive model of ultra-high performance concrete (UHPC) was calibrated using uniaxial tensile test data, thereby obtaining the tensile constitutive relationship of UHPC for flexural strengthening analysis. The accuracy of the established constitutive model was verified by comparing numerical results from finite element simulations with experimental strengthening data. The comparison demonstrated that the finite element calculations effectively replicated the load-displacement response of the test beams, with errors at key characteristic points (e.g., yield load, ultimate load) controlled within 0.49–6.25%, indicating high simulation accuracy.
2. Experimental results showed that the UHPC strengthening layer significantly improves the flexural performance of RC beams. Compared to the unstrengthened beam, the SY_40_ test beam exhibited increases of 40.7% in cracking load, 26.8% in ultimate load, and 25.9% in flexural stiffness. The SY_2 × 12_ test beam showed even greater improvements: 145.2% in cracking load, 67.9% in ultimate load, and 41.4% in flexural stiffness. The UHPC strengthening layer with reinforcement demonstrated superior flexural performance.
3. Full-field strain analysis of the strengthened test beams using digital image correlation (DIC) revealed that the cracking areas of the UHPC strengthening layer and the RC beam did not fully coincide. This phenomenon primarily stems from differences in the bond behavior at the interface between new and old concrete and the resulting stress redistribution. At ultimate failure, only localized horizontal micro-cracks occurred at the interface, with no debonding of the strengthening layer, confirming that surface roughening treatment alone ensures effective collaborative performance at the interface.
4. Finite element analysis indicated that while unreinforced UHPC strengthening layers (e.g., UR_40_, UR_180_) improved the cracking and ultimate loads, they led to significant stress concentration at the beam bottom, sparse distribution of equivalent plastic strain, and a “few but wide” crack pattern. Failure occurred abruptly, and the material’s crack resistance was not fully utilized. In contrast, the reinforced UHPC strengthening layer (UR_2 × 12_) maintained the lowest equivalent plastic strain throughout the loading process, with uniform crack distribution and slow propagation. The load-displacement curve exhibited a prolonged softening stage and a distinct plateau after the peak load, indicating synergistic tension between UHPC and the internal reinforcement. This effectively suppressed crack development in the RC beam, providing sufficient ductile deformation and early warning characteristics before failure. Thus, the reinforced UHPC strengthening method not only enhances the load-bearing capacity but also improves the ductility and controllability of failure.

## Data Availability

The data used to support the findings of this study will be available from the corresponding author upon reasonable request.
